# Supplementary material for: Disparate acidification and calcium carbonate desaturation of deep and shallow waters of the Arctic Ocean
Source: Nat Commun. 2016 Sep 23;7:12821. doi: 10.1038/ncomms12821 (PMC5036158; doi:10.1038/ncomms12821)
Supplement: Supplementary Information — Supplementary Figures 1 - 4, Supplementary Table 1 and Supplementary References [file ncomms12821-s1.pdf]

Supplementary Information

Supplementary Figures

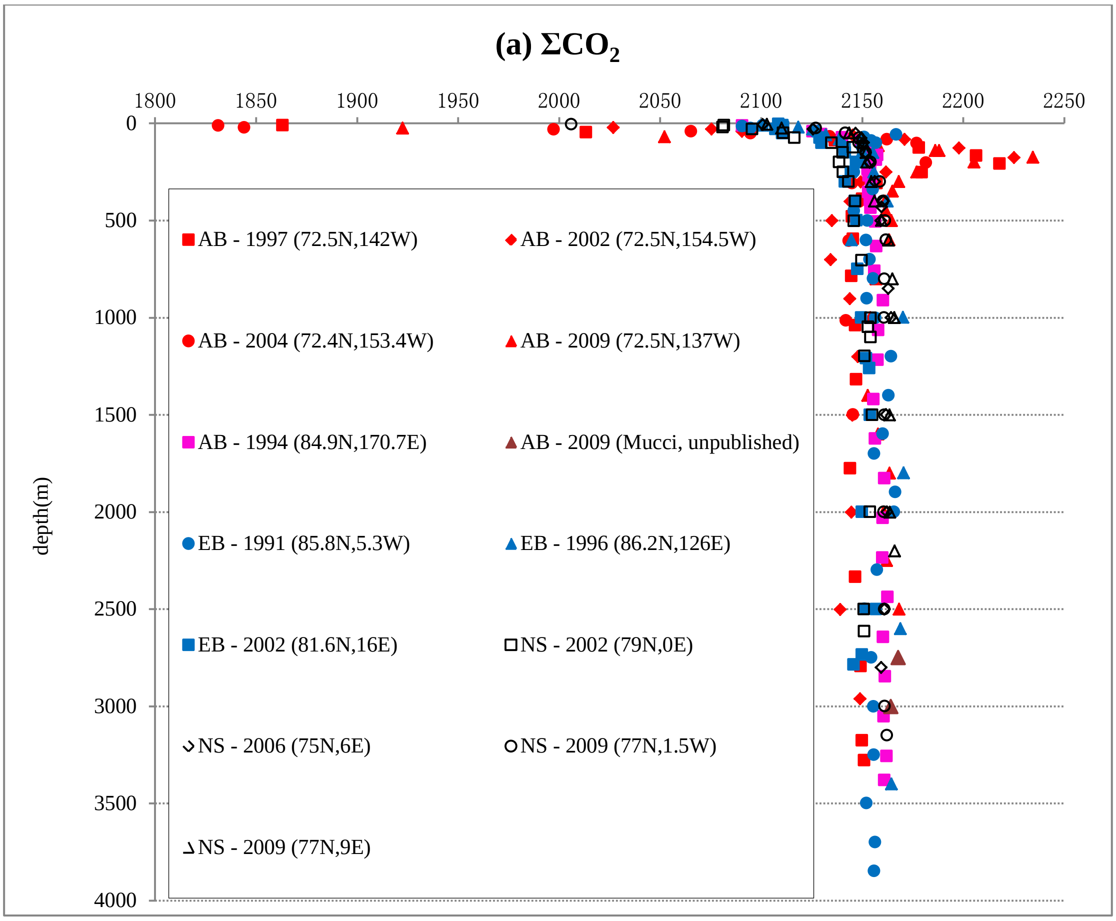

Supplementary Figure 1 $\sum CO_2$ **for Arctic Ocean sites at the Lamont-Doherty Earth Observatory web site.** See http://cdiac.ornl.gov/oceans/LDEO_Underway_Database/ Symbols for the data give the locations by latitude and longitude in parentheses. Data before 2006 are included in the Arctic Ocean data compilation (Jutterstrom et al.[1]) within the carbon dioxide in the Atlantic Ocean (CARINA) data synthesis project (Key et al.[2]). Data after 2006 can be found in the Global Ocean Data Analysis Project version 2 (GLODAPv2) database (Key et al.[3]).

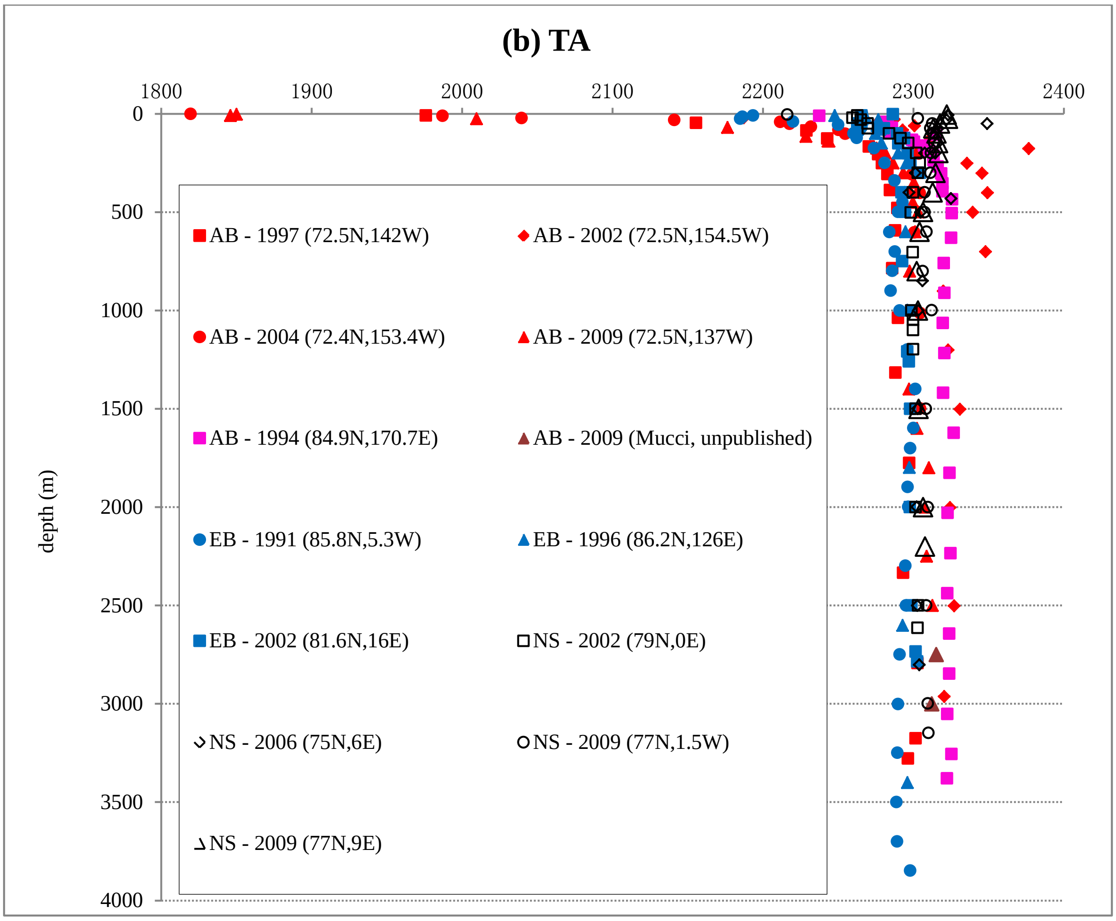

Supplementary Figure 2. ***TA* for Arctic Ocean sites at the Lamont-Doherty Earth Observatory web site.** See http://cdiac.ornl.gov/oceans/LDEO_Underway_Database/ Symbols for the data give the locations by latitude and longitude in parentheses. Data before 2006 are included in the Arctic Ocean data compilation (Jutterstrom et al.[1]) within the carbon dioxide in the Atlantic Ocean (CARINA) data synthesis project (Key et al.[2]). Data after 2006 can be found in the Global Ocean Data Analysis Project version 2 (GLODAPv2) database (Key et al.[3])

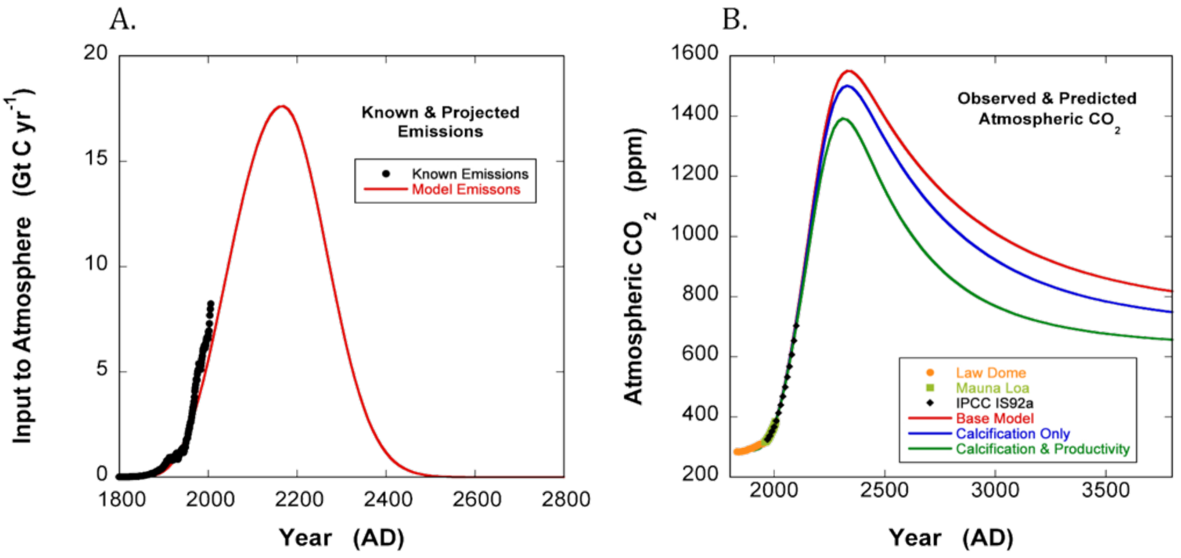

Supplementary Figure 3 Extended IS92a CO_2 emissions scenario in Boudreau et al.[4]. Ignore the blue and green lines in Panel B, as they are not part of the current paper. This scenario is described in detail in Boudreau et al.[4]. It is not the original and dated IS92a scenario. Panel A gives the known emissions from 1800 to 2009 (black dots) and our fit and extension of those emissions into the future (red line), which we label the "extended IS92a" scenario. The emissions curve describes the input of CO_2 to the combined atmosphere- ocean system, hence discounting land uptake; this explains why the model forcing in panel A is slightly lower than the actual emissions data based on estimated fossil fuel emissions. Panel B shows that the emission scenario follows the IPCC IS92a projection for the 21st century, but is entirely consistent with the observed atmospheric CO_2 data from Law Dome and Mauna Loa to year 2010. The extended IS92a then uses a forward Gaussian evolution, which peaks near the year 2250. The total emission is 4025 Gt C over a period of 600 years, which leads to maximum atmospheric CO_2 concentrations of around 1400 ppm. This type of extension is needed because of the long period of integration of our model, i.e., thousands of years.

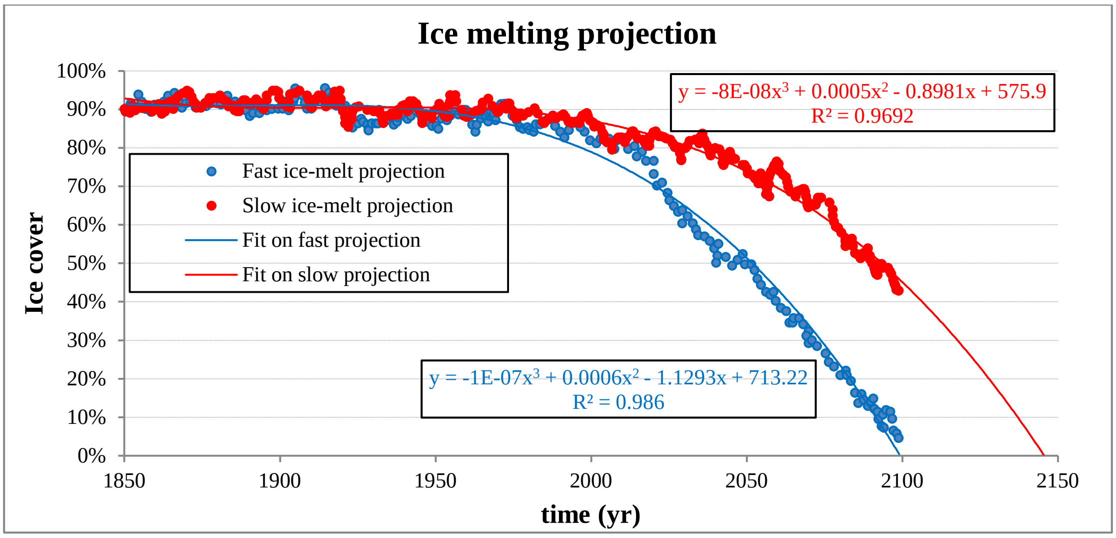

Supplementary Figure 4 Ice melting scenarios tested with our model. Falling ice cover is used to increase gas exchange with the atmosphere with time. The red data are the estimates with “slow” ice melting and the blue data represent “fast” ice melting. The red and blue lines are our best polynomial fits to their respective data. These scenarios are derived from the projections given by Yamamoto et al.[5] and details of these predictions can be obtain in that paper (see their section 3.2). Yamamoto et al. report sea ice extent and we convert that to Ice Cover (%) by dividing by the total ocean area.

Supplementary Tables

Supplementary Table 1 Key parameters, water flows and initial concentration values used in our model.

Quantity	Amerasian Basin	Eurasian Basin
$\sum CO_2$ in S, I and D boxes (μM)	2061.3, 2143.9, 2144.3	2039.1, 2161.8, 2156.1
CA in S, I, D boxes (μM)	2212.8, 2278.1, 2278	2183.8, 2292.7, 2287.7
Freshwater Alkalinity Input (Gmol yr^{-1})	662.8	1988.3
Initial *pH* in S, I, D boxes	8.36, 8.25, 8.18	8.31, 8.21, 8.17
Air-sea CO_2 flux (Gmol yr^{-1})	+500	+500
Initial B, P, B_D (Gmol yr^{-1})	30, 90, 0	10, 30, 0
$U_{T,SP}/U_{T,IA}$, $U_{T,IP}/U_{T,IE}$ (Sv) – not ratios	0.8, 1	6, 2.5
$U_{M,SA}/U_{M,SE}$, $U_{M,DA}/U_{M,DE}$ (Sv) – not ratios	1, 2	1, 2
$U_{T,DBC}$, $U_{M,S}$, $U_{M,I}$ (Sv)	2.5, 2, 2	

Supplementary References

1. Jutterstrom, S. *et al*. Arctic Ocean data in CARINA. Earth Sys. Sci. Data **2**, 71–78 (2010).

2. Key, R. M. *et al.* The CARINA data synthesis project: Introduction and overview, Earth Sys. Sci. Data **2**, 105–121 (2010).

3. Key, R.M. *et al*. Global Ocean Data Analysis Project, Version 2 (GLODAPv2), ORNL/CDIAC-162, ND-P093. Carbon Dioxide Information Analysis Center, Oak Ridge National Laboratory, US Department of Energy (2015), Oak Ridge, Tennessee. doi: 10.3334/CDIAC/OTG.NDP093_GLODAPv2.

4. Boudreau, B. P., Middelburg, J.J., Hofmann, A.F. & Meysman, F.J.R. Ongoing transient in carbonate compensation. *Global Biogeochem. Cycles* **24**, GB4010 (2010).

5. Yamamoto, A., Kawamiya, M., Ishida, A., Yamanaka, Y. & Watanabe, S. Impact of rapid sea-ice reduction in the Arctic Ocean on the rate of ocean acidification. *Biogeosci.* **9**, 2365–2375 (2012).
